# Influences of Initial Stresses on Formation of Shear Bands and Mechanical Properties in Binodal Decomposed Metallic Glass Composites

**DOI:** 10.3390/nano15221725

**Published:** 2025-11-15

**Authors:** Yongwei Wang, Guangping Zheng, Mo Li

**Affiliations:** 1 Collaborative Innovation Center of Steel Technology, University of Science and Technology Beijing, Beijing 100083, China; 2 Department of Mechanical Engineering, Hong Kong Polytechnic University, Hung Hom, Kowloon, Hong Kong, China; mmzheng@polyu.edu.hk; 3 School of Materials Science and Engineering, Georgia Institute of Technology, Atlanta, GA 30332, USA

**Keywords:** initial stress, Binodal decomposed metallic glass composites, free volume theory, shear banding, strength

## Abstract

Structural heterogeneity is useful for improving the plasticity of metallic glasses (MGs) by blocking the propagation of shear bands (SBs). The introduction of a heterogeneous structure often introduces residual stresses, which significantly influences the deformation behaviors of MGs; however, the quantitative impact of residual/initial stresses on shear banding remains unclear. In this work, through finite element models, we demonstrate that residual/initial stresses can promote the initiation of SBs at the interfaces between droplet or particle reinforcements and the matrix in Binodal decomposed metallic glass composites (BDMGCs). These reinforcements do not effectively block the SBs when the fraction of particle reinforcement is very low. We demonstrate that a heterogeneous distribution of initial tensile stresses reduces the strength of BDMGCs, particularly in those containing a homogenous matrix. This profound understanding of the synergistic effects arising from a heterogeneous microstructure and initial stresses could effectively promote the design and optimization of MGs and their composites.

## 1. Introduction

Metallic glasses (MGs), or amorphous alloys, have many excellent mechanical and physical properties in comparison to their coarse-grained crystalline counterparts, which are derived from a disordered atomic structure [1]. However, one major drawback is their limited macroscopic plastic deformability at room temperature due to the formation of highly localized shear bands (SBs) [2]. Enormous efforts have been devoted in recent years to improving the plasticity of MGs. The concept of heterogeneity, including composites, is widely accepted to be useful for improving the plasticity of MGs by blocking SBs [3,4,5,6,7,8,9,10,11,12,13,14,15,16,17,18,19,20,21,22,23,24,25,26]. For instance, the introduction of a second phase or inclusions, surface treatments, or mechanical deformations such as shot peening or grinding in the surface region can induce structural, residual stress or chemical heterogeneity that can contribute to enhanced ductility [25,26,27,28,29,30,31,32,33,34,35,36,37,38,39,40,41,42,43].

Residual stress is usually accompanied by the generated heterogenous structure. For example, varying cooling rates during quick quenching not only create the gradient in free volumes (FVs) but also induce residual stress [28]. In an as-cast MG cylinder, the profile of residual stress exhibits a parabolic form, and the maximum tensile stress at the center is roughly half the maximum compressive stress at the surface [36,37,38]. Shot peening can also introduce surface compressive stress and promote the heterogenous distribution of many SBs [26]. In metallic glass composites (MGCs), particularly in ex situ MGCs, residual stress arises from differences in thermal conductivity and the elastic modulus between the matrix and reinforced phase [36].

In particle-reinforced MGCs, including in situ and ex situ ones, the secondary reinforcement is anticipated to block the propagation of SBs in the matrix and subsequently enhance ductility or toughness [24]. In ex situ MGCs, interfacial reactions take place at particle–matrix interfaces, leading to low wettability and making it challenging to enhance plasticity [6,7,8,9]. In contrast, in in situ MGCs, there are strong atomic-level bonding interfaces between the secondary reinforcement and glassy matrix. When alloys contain a large positive enthalpy of mixing and possess high glass forming ability, metallic glass-forming alloys can separate into two different amorphous phases during solidification [15,16,17,18,19,20,21,22]. An interconnected-type structure (Spinodal decomposition) or a droplet-type (Binodal decomposition) structure can be obtained [20,21]. Heterogeneous microstructures can be at various scales ranging from micro-scales to nano-scales. For the differences in coefficient of thermal expansion and mechanical property, the glassy reinforcement and glassy matrix will both exsit the compressive and tensile re-sidual stresses.

We note that certain Binodal decomposed metallic glass composites (BDMGCs), which have a large fraction of reinforcement, show essentially zero ductility in tension and limited plasticity in compression [22]. Most experimental and theoretical analyses focus on the effects of size and fraction and the morphology of the particles. However, the impact of the spatial residual stresses induced by the mismatch of thermophysical properties on the mechanical properties and shear banding process is rarely quantitatively investigated. This work will quantitatively assess the impact of initial spatial stresses (both compressive and tensile stresses) on the initiation and propagation of SBs in BDMGCs. Additionally, we will explore and investigate the synergistic effects arising from a heterogeneous microstructure and initial stresses. It is essential to develop new theories that effectively incorporate the influences of residual/initial stress in the structural design of MGs or MGCs.

## 2. Materials and Methods

Recently, the effects of compressive residual stress on the hardness of MGs have been extensively studied [25,26,29,30,31,32,33,34,35,36,37,38,39,40,41,42,43,44,45]. For shot-peened MGs, the following argument is proposed: softening via the introduction of SBs and hardening via compressive residual stress [26]. This phenomenon has also been observed in silicate glass. For instance, the exceptional strength of Prince Rupert’s drops is attributed to substantial surface compressive stresses [31]. Moreover, the magnitude of the surface compression developed in tempered glass is significantly higher than that in float glass. However, as the tensile zone of silicate glass is subjected to load, it becomes more brittle. As we know, as the stress equilibrium exist in a stable or metastable system, surface pre-compression should be counterbalanced by an associated subsurface tensile stress field. Consequently, both tensile and compressive residual stresses must be incorporated into models of MGs or MGCs.

**Figure 1:**
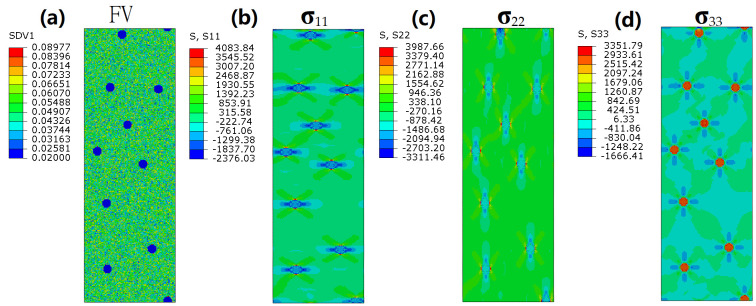
The initial FV state (**a**), residual stress σ_11_ (**b**), residual stress σ_22_ (**c**), and residual stress σ_33_ (**d**) of the MA system with soft particles subjected to hydrostatic pressure.

In this work, we systematically explore how residual/initial stresses (both tensile and compressive) modulate SB dynamics and the mechanical performance of BDMGCs through finite element analysis. BDMGCs have particle/drop-like reinforcements. By assigning the FV and stress values to each element, we establish the initial microstructure (Figure 1a) and spatial stress distribution (Figure 1b). To understand the synergistic effects of residual stress and heterogeneous microstructure on mechanical performance and shear banding, all systems are designed to have the same particle diameter, maintaining an identical volume fraction of the particles. Through finite element models (FEMs) that emphasize the FV parameter as a characteristic quantity, the variation in and evolution of the structure of BDMGCs can be gauged.

To obtain the synergistic effects of initial stress and heterogeneous microstructure on the mechanical properties of BDMGCs with various particle properties on relatively large scales, we employ a continuum approach to model the mechanical behaviors of the BDMGCs that contain submicron-sized particles. The constitutive models that explicitly incorporate FV variations in MGs [25,44,45,46,47,48,49,50,51,52] are used in the FEMs. We assign FV values at the mesh element according to certain spatial distributions, such as those following the drop-like structure (Figure 1). In the FEMs, all elements are square, and the side length is designated as *d* = 0.1 μm. By distributing different FV densities *ρ_M_* and *ρ_p_* in the glassy matrix and particles, we can make a BDMGC heterogeneous structure with spatial patterns. The FV density in a matrix can be determined from statistical distributions, which are obtained via the transformations of the beta distribution, 0.04 × *Beta*(50,50) + 0.03, 0.04 × *Beta*(1,1) + 0.03, or 0.04 × *Beta*(0.1,0.1) + 0.03, which is labeled as matrix A, B, or C, respectively. The FV distribution in matrix A resembles a truncated Gaussian, B a random distribution, and C a bimodal distribution, all of which have a mean FV value of 0.05 [49]. The range of FVs in these matrices is limited from 0.03 to 0.07. The BDMGC samples, which consist of matrices A, B, and C, are labeled as systems MA, MB, and MC, accordingly. The FV values *ρ_p_* for the particle range from 0.02 to 0.10, so we can see how the varying mechanical properties of reinforcements affect the overall mechanical properties of BDMGCs with an FV change from a small *ρ_p_* (=0.02), or “hard” particles, to a larger *ρ_p_* (=0.10), or “soft” particles. Particle diameter *D* can be measured using the number of elements across the particle, so *D* is expressed as a multiple of *d*. Here *D* is taken as 8, and the total volume fraction of 10 particles is about 2.3%.

Figure 1a shows the MA BDMGC system containing soft particles. An isolated particle with diameter *D* is compressed by hydrostatic pressure *P* (in GPa). The tensile stress distribution of the matrix in the spherical coordinate system is as follows:

(1) $$\sigma_{r}=P\frac{D^{3}}{8r^{3}}$$

(2) $$\sigma_{\theta }=\sigma_{\varphi }=P\frac{D^{3}}{16r^{3}}$$

where *D* is the particle diameter, and *r* is the distance from the center of the particle [53]. Based on Saint-Venant’s principle, the range or diameter of residual stress distribution is 5 times the particle diameter. In the plane strain model, the compressive stresses of inner isolated particles are as follows:

(3) $$\sigma_{r}=\sigma_{\theta }=P$$

(4) $$\sigma_{z}=2\mu P$$

while the tensile stresses in the matrix are as follows:

(5) $$\sigma_{r}=(1-\;\frac{25D^{2}}{4r^{2}})\frac{P}{24}$$

(6) $$\sigma_{\theta }=(1+\frac{25D^{2}}{4r^{2}})\frac{P}{24}$$

(7) $$\sigma_{z}=2\mu \frac{P}{24}$$

The principal stresses I, II, and III are illustrated in Figure 1b–d under a Cartesian coordinate system, respectively.

As shown in previous work, the choice of these parameters allows us to carry out a systematic and quantitative investigation of the initial stress effect on shear banding and the synergistic effects of initial stress and heterogeneous microstructure on mechanical properties. To obtain the mechanical properties of all samples, we use an elastoplastic constitutive model that incorporates FV as an internal state variable. The deformation strain in this model includes an elastic and a plastic part:

(8) $$\varepsilon_{ij}=\varepsilon_{ij}^{el}+\varepsilon_{ij}^{pl}$$

where $\varepsilon_{ij}^{el}$ is the elastic strain. We obtain the plastic strain from the plastic flow equation:

(9) $$d\varepsilon_{ij}^{pl}=d\lambda \frac{\partial g}{\partial \sigma_{ij}}$$

where *g* is the plastic potential function.

(10) $$g(\sigma_{ij})=b^{\prime }I_{1}+\sqrt{3J_{2}}-K$$

where λ is the plastic deformation parameter related to FV change, $\sigma_{ij}$ is the Cauchy stress, $I_{1}$ is the first invariant of the stress tensor $\sigma_{ij}$, and $J_{2}$ is the second invariant of the deviatoric stress. The effective stress and increase in equivalent plastic strain are obtained through the following relations:

(11) $$\sigma_{DP}=a^{\prime }I_{1}+\sqrt{3J_{2}}$$

(12) $$d\varepsilon_{eff}^{pl}=\sqrt{(2/3)d\varepsilon_{ij}^{pl}d\varepsilon_{ij}^{pl}}$$

where $a^{\prime }$, $b^{\prime }$, and K are constant, and $a^{\prime }=b^{\prime }$ for the associated flow rule. The plastic strain is a function of FV production via the following relation:

(13) ε˙effpl=2fexp−αv*v¯fexp−ΔGmkBTsinhτΩ2kBT

(14) ∂v¯f∂t=v*fexp−αv*v¯fexp−ΔGmkBT2αkBTv¯fScoshτΩ2αkBT−1−1nD+κ∇2v¯f

where ${\bar{v}}_{f}$ is the mean FV; α a geometrical factor close to 1; $v^{*}$ the hard-sphere volume of the atom; $k_{B}$ the Boltzmann constant; Ω the atomic volume; τ the equivalent shear stress; $\Delta G^{m}$ the activation energy; f the frequency of atomic vibration; T temperature; $n_{D}$ the number of atomic jumps needed to annihilate a free volume equal to $v^{*}$ which ranges between 3 and 10; $S=\frac{E}{3(1-\mu )}$, where E is Young’s modulus and μ Poisson’s ratio; and κ a free volume gradient coefficient.

Equations (8)–(14) prescribe the constitutive relationship for the MG. Whether the deformation behavior is homogeneous or inhomogeneous is not explicitly distinguished. The FV and the stress field will be computed using a finite element method. Given the history of applied load, we aim to calculate the displacements, strains, and stresses satisfying the following governing equations:

(15) $$\frac{\partial \sigma_{\mathrm{ji}}}{\partial x_{i}}+\frac{\partial \rho \nu_{i}}{\partial t}+\rho f_{i}=0$$

(16) $$\varepsilon_{\mathrm{ij}}=\frac{1}{2}(u_{i,j}+u_{j,i})$$

where the boundary condition is as follows: ${\sigma^{*}}_{ij}n_{j}=t_{i}$. Here we assume that the body force is zero, i.e., $f_{i}$ = 0, and ignore the density change rate, i.e., $\frac{\partial \rho }{\partial t}$ = 0. Based on the variation principle, we transfer the partial differential equations of the force balance Equation (15) and their boundary conditions into the weak form:

(17) $$\int_{V}\sigma_{\mathrm{ij}}\delta \varepsilon_{ij}dV+\int_{\partial V}{\sigma^{*}}_{ij}n_{j}\delta v_{i}dS=0$$

In the FEM, the displacement field at an arbitrary point within the solid will be specified by interpolating between nodes of mesh through the shape function *N*. Substituting the interpolated fields into the virtual work equation, we find the following:

(18) $$\int_{V}\sigma_{ij}(\Delta \varepsilon_{\mathrm{kl}}(u_{i}^{a}))\frac{\partial N^{a}}{\partial x_{j}}dV+\int_{V}\frac{\partial \Delta \sigma_{ij}}{\partial \Delta \varepsilon_{\mathrm{kl}}}\frac{\partial N^{b}}{\partial x_{i}}\frac{\partial N^{a}}{\partial x_{j}}\Delta u_{i}^{a}dV+\int_{\partial V}{\sigma^{*}}_{ij}n_{j}N^{a}dS=0$$

We can therefore rewrite the virtual work equation in a system of linear equations. The material-related parameter pertaining to the constitutive models, i.e.,

(19) $$D_{ijkl}^{ep}=\partial \Delta \sigma_{ij}/\partial \Delta \varepsilon_{kl},$$

is implemented in ABAQUS 6.13 finite element software through a UMAT subroutine [47,48,49]. In the implicit integration scheme, applying the backward Euler method to the flow Equation (13) and FV evolution Equation (14), we get the following:

(20) $$\sigma_{DP,(n+1)}^{trial}-\sigma_{DP,(n)}-\Delta \sigma_{DP}-(Kab+3G)\frac{1}{\sqrt{\frac{2}{9}b^{2}+1}}2\Delta t\exp(-\;\frac{1}{{\bar{v}}_{f(n)}+\Delta {\bar{v}}_{f}})\sin h(\frac{\sigma_{DP(n)}+\Delta \sigma_{DP}}{\sigma_{0}})=0$$

(21) $$\Delta {\bar{v}}_{f}\;-\;\frac{\Delta t}{\alpha }\exp(\;-\;\frac{1}{{\bar{v}}_{f(n)}+\Delta {\bar{v}}_{f}})[\frac{1}{({\bar{v}}_{f(n)}+\Delta {\bar{v}}_{f})\beta S}(\cosh(\frac{\sigma_{DP(n)}+\Delta \sigma_{DP}}{\sigma_{0}})\;-\;1)\;-\;\frac{1}{n_{D}}+\kappa \nabla^{2}{\bar{v}}_{f(n)}]=0$$

The initial guess solution ($\Delta {{\bar{v}}_{f}}^{0},\Delta {\sigma_{DP}}^{0}$) is taken as the solution from the previous time step. In the Newton–Raphson method, the solution ($\Delta {{\bar{v}}_{f}}^{k},\Delta {\sigma_{DP}}^{k}$) at the ***n***th step will be updated for the calculation ($\Delta {{\bar{v}}_{f}}^{k+1},\Delta {\sigma_{DP}}^{k+1}$) in the next step. The trial stress tensor is $\sigma_{DP,(n+1)}^{trial}$. The material stiffness matrix $D_{ijkl}^{ep}$ can also be updated. By solving the system of linear equations of FV evolution and the strain and stress under a given external load, we can obtain the mechanical properties of BDMGCs containing various particles and initial stress. The material properties of bulk Zr_41.25_Ti_13.75_Ni_10_Cu_12.5_Be_22.5_ MG are used [48]. The model systems have a total of 30,000 regular elements and the periodic boundary conditions in our FEM. Plane strain tensile loadings are applied with an effective strain rate of 0.1/s.

## 3. Results

**Figure 2:**
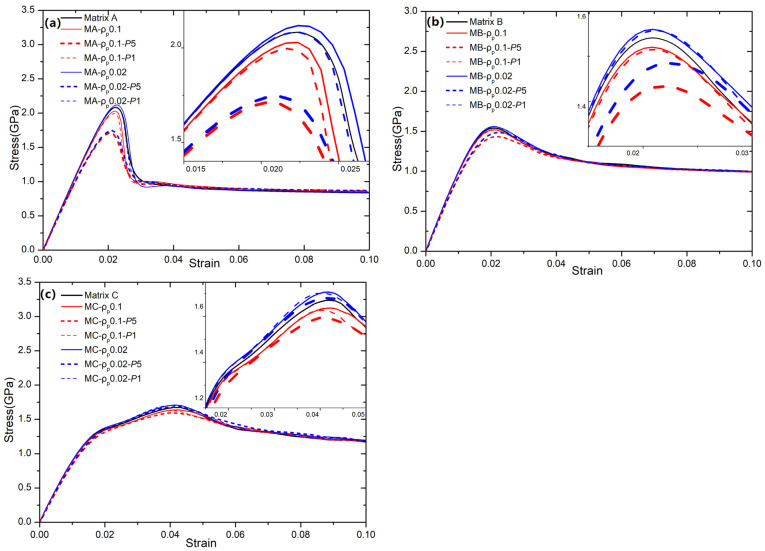
The stress–strain curves for the systems MA (**a**), MB (**b**), and MC (**c**) with soft particles *ρ_p_* = 0.10 (red line) and hard particles *ρ_p_* = 0.02 (blue line). The dashed and solid lines indicate systems with and without initial residual stresses, respectively. The black line denoting “Matrix” represents the monolithic MG.

The tensile stress–strain relationships of all systems with various particle characteristics and varying residual/initial stresses are shown in Figure 2. In the following, we will categorize 21 types of samples based on three statistical FV distributions of the matrix, incorporating three types of residual stress and two types of particles. For example, “MA-*ρ_p_*0.02-*P*5” indicates that the BDMGC system comprises matrix A and hard particles (*ρ_p_* = 0.02) subjected to a hydrostatic pressure of *P* = 5 GPa. Meanwhile, “MA-*ρ_p_*0.02” signifies that the system is devoid of any initial stress. In general terms, compared with the MG matrix, the hard reinforcement increases strength, while the soft one results in a reduction in strength. The results corroborate that an FV density of 0.02 (or harder particles) enhances the strength of the composite (as illustrated by the blue lines in Figure 2). Conversely, softer particles with an FV density of 0.10 lead to a reduction in composite strength (as illustrated by the red lines in Figure 2) as compared to that in the monolithic sample (as represented by the black lines in Figure 2). This is evidenced by the changes observed in mechanical strength such as yield stress and peak stress. The yield stress is determined by the 0.2% strain offset method. The peak stress is taken at the first occurrence of the maximum stress. The flow stress is the asymptotic stress value at the large deformation strain. In Figure 2, the flow stresses of all systems with varying initial stresses remain the same as compared to the one without residual stress. The increased residual stresses (containing the compressive and tensile stresses) lead to a greater reduction in peak stress. As illustrated in Figure 2, the decrease in peak stress is alleviated as the heterogeneity of the matrix increases, which corresponds to the statistical variance of FV [49].

**Figure 3:**
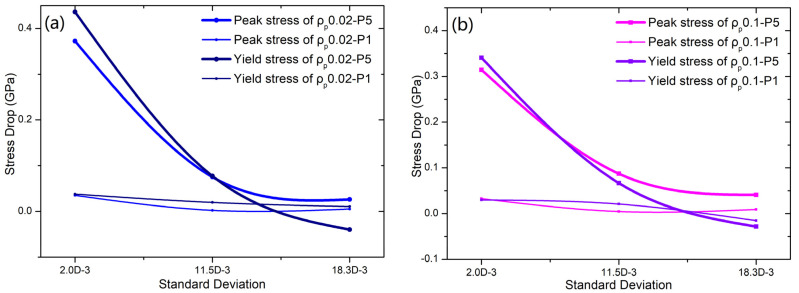
The mechanical strength reductions (yield stress and peak stress) in BDMGC samples with hard particles (*ρ_p_* = 0.02) (**a**) and soft particles (*ρ_p_* = 0.10) (**b**), influenced by initial stresses, are observed in relation to matrix heterogeneity (standard deviation of matrix).

The standard deviations (SDs) of matrices A, B, and C are 0.002, 0.0115, and 0.0183, respectively. Comparing the peak stress of the samples “MA-*ρ_p_*0.02-*P*5” and “MA-*ρ_p_*0.02”, it is clearly observed that the peak stress decreases from 2.0836 GPa to 1.7463 GPa. For the samples “MB-*ρ_p_*0.02-*P*5” and “MB-*ρ_p_*0.02”, their peak stress decreases from 1.560 GPa to 1.4850 GPa. The peak stress of samples “MC-*ρ_p_*0.02-*P*5” and “MC-*ρ_p_*0.02” decreases from 1.7066 GPa to 1.6806 GPa. The yield stress reductions for samples “MA-*ρ_p_*0.02”, “MB-*ρ_p_*0.02”, and “MC-*ρ_p_*0.02” are 0.4362 GPa, 0.07745 GPa, and 0.003976 GPa, respectively. As shown in Figure 3a, one can see a reduction in the strength (yield and peak stresses) of all samples as the standard deviation of the matrix increases. As particles are subjected to a hydrostatic pressure of ***P*** = 1GPa, the yield stress reductions for samples “MA-*ρ_p_*0.02”, “MB-*ρ_p_*0.02”, and “MC-*ρ_p_*0.02” are 0.03776 GPa, 0.01965 GPa, and 0.01063 GPa, respectively. This trend becomes increasingly pronounced as the residual stress increases. For the system with soft particles (*ρ_p_* = 0.1), the trend in strength reduction resembles that in hard particles, as shown in Figure 3. As particles are subjected to a hydrostatic pressure of ***P*** = 5 GPa, the reductions in peak (yield) stress for samples “MA-*ρ_p_*0.02”, “MB-*ρ_p_*0.02”, and “MC-*ρ_p_*0.02” are 0.3144 GPa, 0.08725 GPa, and 0.04078 GPa (0.34089 GPa, 0.06675 GPa, and −0.02845 GPa), respectively.

## 4. Discussion

**Figure 4:**
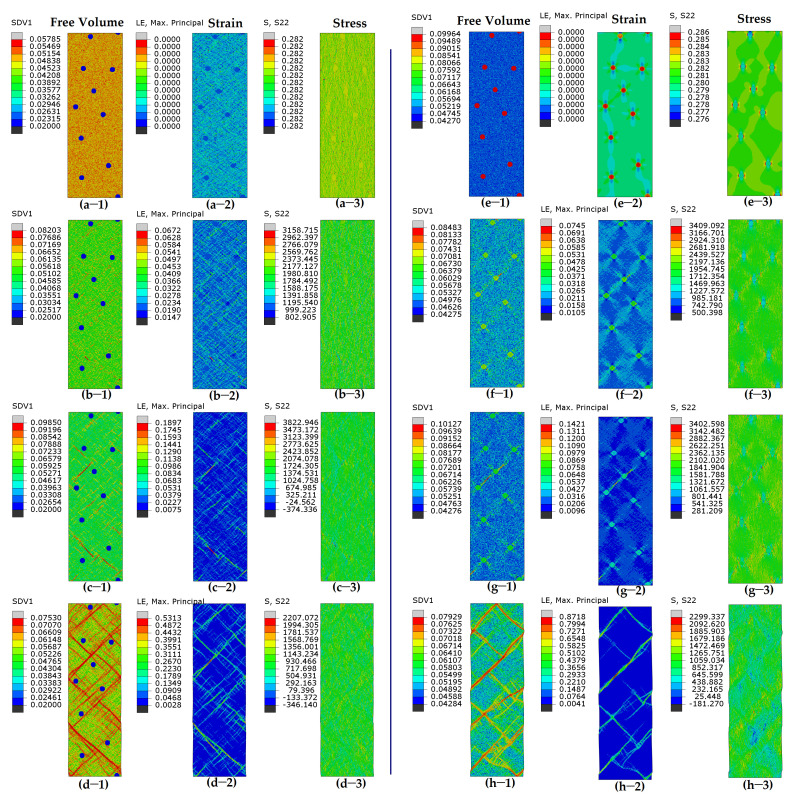
Contour plots for free volume (-1), shear strain (-2), and shear stress (-3) for MA system with (**a**–**d**) hard particles (*ρ_p_* = 0.02) or (**e**–**h**) soft particles (*ρ_p_* = 0.1) under varying tensile strains. Color scheme is shown on left of each figure.

**Figure 5:**
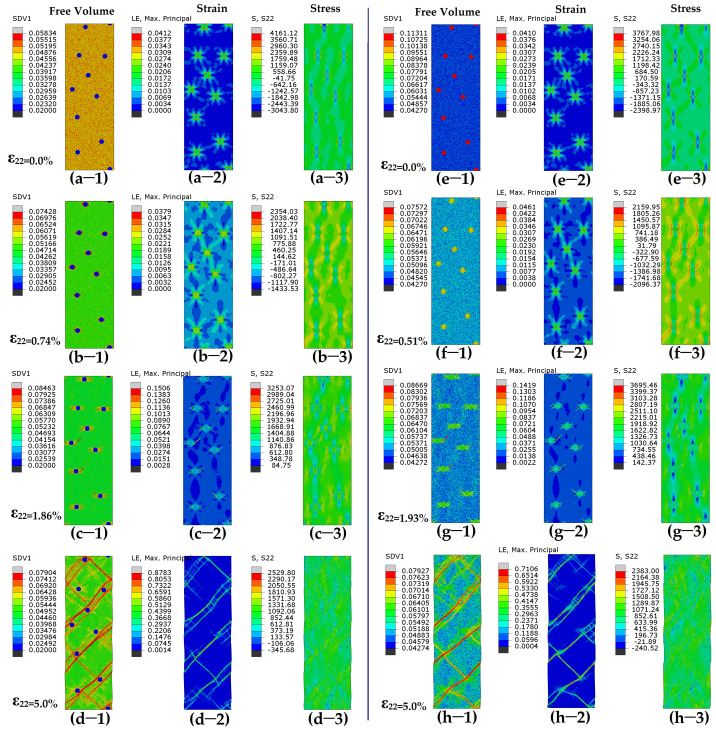
Contour plots for free volume (-1), shear strain (-2), and shear stress (-3) for MA system with (**a**–**d**) hard particles (*ρ_p_* = 0.02) or (**e**–**h**) soft particles (*ρ_p_* = 0.10) under varying tensile strains. MA system contains initial stresses before deformation. Color scheme is shown on left of each figure.

The presence of initial stresses (initial compressive and tensile stresses) contributes to a reduction in strength (peak stress), which suggests the emergence of new deformation mechanisms. This synergistic effects of residual/initial stress and heterogeneous microstructure on mechanical performance will be discussed later. To investigate the new deformation mechanism, the spatial FV distributions, strain, and stress of all systems under various applied strain states are examined to analyze the behaviors of local shear, as shown in Figure 4 and Figure 5. In Figure 4, the samples “MA-*ρ_p_*0.02” and “MA-*ρ_p_*0.1” exhibit deformation in the absence of initial stress, while the deformation of samples “MA-*ρ_p_*0.02-*P*5” and “MA-*ρ_p_*0.1-*P*5” containing initial stresses is shown in Figure 5. Figure 4a shows that for the sample “MA-*ρ_p_*0.02” containing hard particles, SBs usually initiate at the matrix and then propagate into mature SBs along the direction of the maximum effective stress (Figure 4b). The location for the initiation of SBs is random due to the random distribution of particles. The stoppage and obstacles of SBs occur when they encounter hard particles, leading to an increase in strength. During the process of the propagation of SBs, some but not all SBs will meet, and they are subsequently stopped by hard particles (Figure 4c,d). A higher fraction of particles increases the likelihood of encountering obstacles for SBs. For the sample “MA-*ρ_p_*0.1” containing soft particles (Figure 4e), deformation first happens in the soft particle zone. Minor SBs will quickly initiate and nucleate in glassy droplets. However, they are trapped and restricted on the droplets (Figure 4f). The main SBs usually initiate at the middle region between two soft particles whose center line is about 45° off the loading axis (Figure 4g). The SBs will propagate and pass through soft particles. Several main SBs will connect and then traverse through the cross-section, as shown in Figure 4h.

In the following, we present a detailed examination of BDMGCs with initial stresses. The initial stresses and FV distribution of samples “MA-*ρ_p_*0.02-*P*5” and “MA-*ρ_p_*0.1-*P*5” are illustrated in Figure 5a,e, respectively. For sample “MA-*ρ_p_*0.02-*P*5”, the initiation point of SBs is at the interfaces between the matrix and hard particles (Figure 5b). The total strain (0.74%) of SB nucleation is significantly smaller than that (2.46%) of the sample without initial stresses. With further deformation, the nucleus of SBs will propagate and develop into mature SBs along the direction of the maximum effective stress (Figure 5c). The SBs will intersect and interconnect with each other (Figure 5d). For sample “MA-*ρ_p_*0.1-*P*5”, deformation first occurs in the soft particle zone under low strain (Figure 5f). However, the initial stress can expand the soft zone (Figure 5g), and then minor SBs will propagate along the direction of the maximum effective stress in the matrix (Figure 5h). In samples “MA-*ρ_p_*0.02-*P*5” and “MA-*ρ_p_*0.1-*P*5”, since the initiation point and direction of SBs are identical, mature SBs appear nearly indistinguishable from one another.

**Figure 6:**
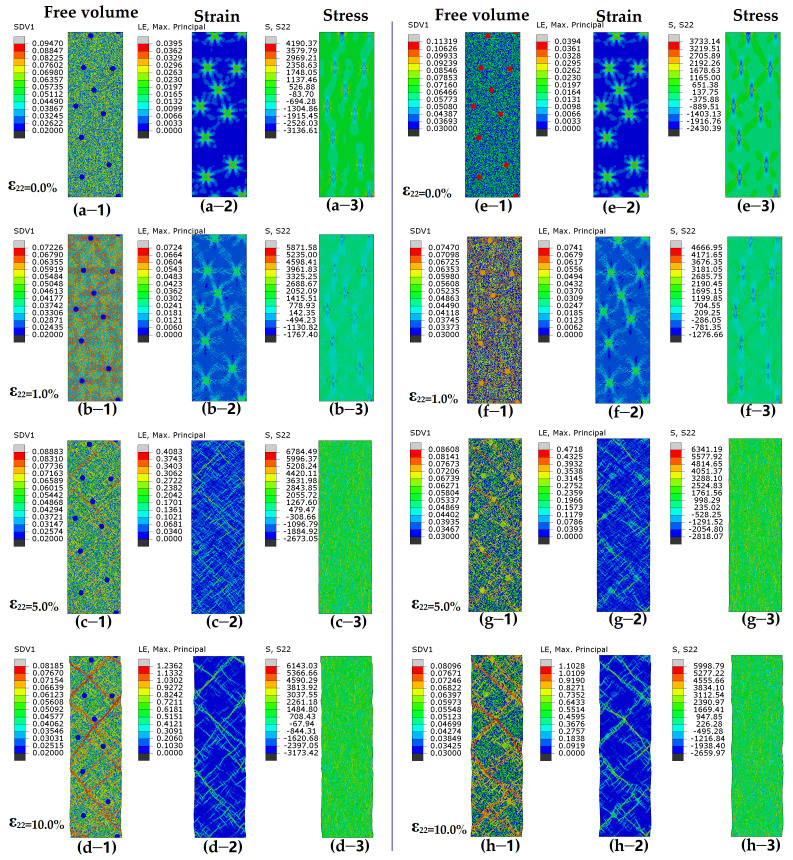
Contour plots for free volume (-1), shear strain (-2), and shear stress (-3) for MC system (**a**–**d**) with hard particles (*ρ_p_* = 0.02) or (**e**–**h**) soft particles (*ρ_p_* = 0.10) under varying tensile strains. MC system contains initial stresses before deformation. Color scheme is shown on left of each figure.

For the systems with a tough matrix (larger statistical variance), the initial stress and FV distribution of samples “MC-*ρ_p_*0.02-*P*5” and “MC-*ρ_p_*0.1-*P*5” are illustrated in Figure 6a,e, respectively. For sample “MC-*ρ_p_*0.02-*P*5”, the initiation point of SBs is at the interfaces between the matrix and hard particles, as shown in Figure 6b. As deformation continues, the deformed regions are still restricted to their original location, while new deformed areas with higher FV values emerge elsewhere. Lots of localized deformation zones form into minor SBs in Figure 6c. The deformation bands in matrix C appear irregular and jagged, unlike the smooth and straight ones in matrix A. Short minor SBs begin to intertwine and linkup, and this then leads to the formation and propagation of mature SBs across the sample, as shown in Figure 6d. For sample “MC-*ρ_p_*0.1-*P*5”, deformation first occurs in the soft particle zone under low strain. However, minor SBs in the soft zone will be restricted by the toughened matrix in Figure 6f. For the MC system with a larger range of FV distribution, the points with a large FV will also be good for the initiation of minor SBs. The initial residual stress cannot result in the easy expansion of minor SBs, as shown in Figure 6f. The nuclei of minor SBs will propagate as rugged, zigzag SBs, with a few maturing and propagating along the direction of the maximum effective stress in the matrix (Figure 6h).

The peak stress of the samples “MA-*ρ_p_*0.02-*P*5”, “MA-*ρ_p_*0.02-*P*1”, and “MA-*ρ_p_*0.02” shows a clear decrease as initial stresses (both compressive and tensile stresses) increase. Similarly, in systems MB and MC with soft or hard particles, the peak stress also decreases as the initial equivalent stresses increase. The standard deviation (SD) or heterogeneity of the matrix can enhance toughness and strength within a given type of matrix. The interplay between initial stresses and the significant heterogeneity of the matrix, coupled with the characteristics of particles, indicates the potential emergence of novel deformation mechanisms. The synthetic effect of initial spatial stresses and heterogeneity results from the SBs induced by temporal and spatial stress localization. The initial spatial distribution of the tensile stresses may put the disordered atoms in a high-energy state according to free volume theory, and consequently, small deformation can store enough energy and stress to trigger microscopic rearrangements, which contribute small local macroscopic shearing. The nucleation and propagation of SBs in the matrix are influenced by heterogeneity (the statistical variance of FV distribution). It is observed that shear banding initiates predominantly in regions of higher tensile stress and larger FV, and SBs grow along the direction of the maximum shear stress during deformation.

## 5. Conclusions

Using finite element modeling, we obtain the mechanical properties of BDMGCs with different spatial stresses and various properties of particle reinforcement, quantitatively investigating their influences on the mechanical properties and mechanisms of shear banding. The heterogeneous distribution of initial tensile stresses reduces the strength of BDMGCs, particularly in those containing a homogenous matrix. The spatial distribution of residual/initial stresses can tune and even determine the nucleation of SBs if the residual/initial tensile stress is high enough. We also show that the heterogeneous microstructure of BDMGCs can influence the propagation of SBs. By elucidating the micro-scale mechanics of BDMGC systems, we provide foundational insights for designing BDMGCs with spatial stresses and heterogeneous microstructures. The quantitative framework demonstrates that BDMGCs can be optimized by tuning heterogeneous stresses and microstructures.

## Data Availability

The raw data supporting the conclusions of this article will be made available by the authors on request.
